# Pathogenesis and Therapy of Oral Carcinogenesis

**DOI:** 10.3390/ijms25126343

**Published:** 2024-06-08

**Authors:** Marko Tarle, Ivica Lukšić

**Affiliations:** 1 Department of Maxillofacial Surgery, Dubrava University Hospital, 10000 Zagreb, Croatia; luksic@kbd.hr; 2 School of Dental Medicine, University of Zagreb, Gundulićeva 5, 10000 Zagreb, Croatia; 3 School of Medicine, University of Zagreb, Šalata 3, 10000 Zagreb, Croatia

Oral squamous cell carcinoma (OSCC) is the most common malignant tumor of the head and neck with an extremely poor five-year survival rate of approximately 50 to 55%, despite significant advances in diagnostic and therapeutic procedures over the past three decades [1,2,3]. According to GLOBOCAN data (IARC, WHO), 389,846 new cases of lip and oral cavity cancer were diagnosed in 2022, while 188,438 people died from this disease. There is a slight upward trend in incidence and mortality, particularly in Asian countries, where this type of cancer ranks seventh in incidence and eighth in mortality in men and fourth in men under 40 years of age [4]. Europe ranks second in the world in terms of incidence and mortality, with OSCC being the thirteenth leading cause of death from malignant disease in men [5]. Although the oral cavity is easily accessible for clinical examination, the poor prognosis is partly due to the fact that more than 50% of OSCC is diagnosed at an advanced stage of disease (stages III and IV), and more than 40% of patients have regional metastases at the time of diagnosis. The late diagnosis of the disease, as well as numerous other problems faced by patients and healthcare professionals, affect patients’ quality of life and survival [6]. OSCC patients have the highest suicide rate among oncology patients, which is 12 times higher than in the general population [7]. In addition, 15% of patients are no longer in follow-up care after surgery, about 35% continue to consume tobacco products, and 16.7% consume alcohol [8,9]. Chronic alcohol and tobacco consumption is responsible for the occurrence of other primary tumors (head and neck, lung, colon, liver, pancreas) in 17.8% of patients in the early stages of the disease [10]. Worryingly, the incidence of OSCC is increasing in the younger population, particularly in white women under the age of 40 who do not smoke or drink alcohol. Although high-risk human papillomavirus type 16 (HPV) infection is responsible for more than 75% of squamous cell carcinomas of the oropharynx, only about 2% of oral cavity carcinomas are caused by HPV [4,6,11]. This suggests the need to investigate other, rarer causes that may be associated with the development of OSCC, such as autoimmune diseases, infectious diseases, immunosuppressive conditions, and familial cancer syndromes (Fanconi anemia, xeroderma pigmentosum, Li-Fraumeni syndrome, Bloom syndrome, ataxia-telangiectasia, and Cowden syndrome), as well as drugs that modulate the immune system [12,13,14,15,16].

The primary treatment for OSCC is surgery, but it is difficult to achieve adequate resection margins due to the complex anatomy and high rate of positive margins (12–30%). Detection of lymph node metastases is an additional challenge due to the risk of occult disease, and there are currently no real-time intraoperative imaging techniques to differentiate between healthy and tumor tissue [2,6,17]. All of this points to the complexity of this cancer, which affects all aspects of health—physical, psychological, and social. A multidisciplinary approach and the presentation of all patients to tumor boards in specialized and well-equipped centers are necessary to improve treatment outcomes [18,19].

This Special Issue provides important insights into the latest research on the pathogenesis and treatment of OSCC. The research included in this issue analyzes in detail the complex molecular mechanisms underlying the development and progression of OSCC and identifies novel biomarkers and therapeutic targets. Through their comprehensive approach, the authors of these articles contribute significantly to our understanding and open up new possibilities for diagnosis and treatment.

OSCC develops from the epithelium of the oral cavity mucosa under the influence of genetic, epigenetic, and environmental factors, as well as precancerous lesions such as leukoplakia and erythroplakia. Key features of cancer include maintenance of proliferation, evasion of growth suppressors, resistance to cell death, immortality, angiogenesis, invasion, metastasis, deregulation of energy balance, and evasion of destruction by the immune system [20,21]. EGFR plays a key role in oral carcinogenesis by regulating signaling pathways such as MAPK and PI3K and by promoting proliferation and activation of the oncogene cyclin D1 [22]. Overexpression of EGFR is associated with poor prognosis in many cancers. Cívico-Ortega, J.L. et al. conducted an interesting systematic review and meta-analysis to evaluate the prognostic and clinicopathologic significance of EGFR overexpression in OSCC. The study found that EGFR overexpression in OSCC patients was significantly associated with poorer overall survival, higher likelihood of neck lymph node metastasis, and higher risk of poorly differentiated tumors. These results suggest that EGFR may be a valuable prognostic biomarker for OSCC [23]. Numerous studies indicate that factors such as epidermal growth factor, hydrogen, UV radiation, and ionizing radiation can cause translocation of EGFR to the nucleus, where it interacts with numerous transcription factors (cyclin D1, BCRP, Aurora kinase A, COX-2, gene regulator c-Myc, iNOS) and influences the activation of genes involved in cell proliferation, tumor progression, and DNA repair [24]. The proteins p53 and cyclin D1 are of critical importance in OSCC, with p53 mutations often leading to overexpression in the early stages of carcinogenesis, while overexpression of cyclin D1 regulates the cell cycle and is associated with aggressiveness and tumor progression. These biomarkers have significant potential for prognostic evaluation and therapeutic targets in OSCC [25,26]. Tarle, M. and colleagues conducted a retrospective study that included immunohistochemical analysis of nuclear EGFR (nEGFR) expression and other markers such as Ki-67, p53, cyclin D1, and ABCG2 in samples of healthy oral mucosa, premalignant changes, and OSCC. The results showed that overexpression of nEGFR was associated with poorer overall survival in OSCC patients, suggesting that nEGFR may be an important prognostic biomarker in oral carcinogenesis. It was also shown that p53 is significantly more expressed in patients with premalignant changes and OSCC compared to healthy oral mucosa and that its expression is associated with perineural invasion (PNI). Cyclin D1 expression was significantly higher in patients with premalignant changes and OSCC compared to healthy oral mucosa, suggesting its role in disease progression [27].

Cetuximab, an antibody targeting EGFR, can be combined with inhibitors of PI3K/Akt signaling to enhance the effect [28]. In this Special Issue, a study by Kleszcz, R. et al. was published in which the combination of an inhibitor of the Wnt/β-catenin signaling pathway (PRI-724) with vismodegib, erlotinib, or HS-173 was examined to investigate their effects on squamous cell carcinomas of the head and neck. The results showed that these combinations synergistically inhibit cell viability, induce apoptosis, and reduce cell migration. The decrease in the expression of cancer stem cell markers such as POU5F1 was particularly significant [29]. Lysyl oxidase (LOX) promotes extracellular matrix maturation and tumor invasiveness, while its propeptide (LOX-PP) inhibits tumor growth. The G473A polymorphism in the LOX gene reduces the tumor suppressor function of LOX-PP and is associated with a higher risk of various types of cancer [30]. In a study, Peymanfar, Y. et al. investigated the influence of the G473A polymorphism of the lysyl oxidase gene (LOX) on the development of OSCC in humans and mice. The results showed that the G473A polymorphism exacerbates the development of OSCC and is associated with a higher frequency and severity of lesions in carriers of this genetic variant. This study suggests that the G473A polymorphism may be an important biomarker for susceptibility to OSCC [31]. Cholesterol also plays an important role in carcinogenesis as it is used for membrane biogenesis and cell signaling. Cholesterol-lowering drugs have shown tumor suppressive effects in OSCC, suggesting that cholesterol plays an important role in the pathogenesis of this cancer [32,33]. Chan, N.N. et al. investigated the role of cholesterol in regulating the localization of caveolin-1 (CAV1) and cell migration in OSCC. The results showed that the addition of cholesterol caused a polarization of cell morphology, a localization of CAV1 at the posterior margin, and a promotion of cell migration. In addition, high expression of membrane CAV1 in tissue samples was associated with OSCC recurrence, suggesting that cholesterol is important for OSCC progression [34].

We hope that these publications will provide useful information, stimulate further discussion, and guide future research. Understanding oral carcinogenesis, the tumor itself, and its microenvironment is crucial for early detection, appropriate treatment, and monitoring of the disease. Only with an integrated approach and a detailed understanding of the molecular mechanisms can we improve outcomes for OSCC patients.
